# The complete mitochondrial genome of *Sarcophaga gracilior* (Diptera: Sarcophagidae)

**DOI:** 10.1080/23802359.2021.1927871

**Published:** 2021-05-20

**Authors:** Xiangyan Zhang, Haojie Tang, Jianan Dong, Lipin Ren, Yadong Guo

**Affiliations:** a Department of Forensic Science, School of Basic Medical Sciences, Central South University, Hunan, Changsha, China; b School of Architecture and Art, Central South University, Hunan, Changsha, China

**Keywords:** Mitochondrial genome, *Sarcophaga gracilior*, phylogenetic analysis

## Abstract

*Sarcophaga gracilior* Chen, 1975 (Diptera: Sarcophagidae) plays a significant role in epidemiology and medicine. In this study, we first report the complete mitochondrial genome (mitogenome) of *S. gracilior*. This mitogenome was 15,534 bp in length (GenBank No. MW531675), comprising 13 protein-coding genes (PCGs), two ribosomal RNAs (rRNAs), 22 transfer RNAs (tRNAs), and a non-coding control region. The arrangement of genes was identical to that of ancestral metazoan. Nucleotide composition revealed a strong A + T bias, accounting for 76.7% (A 39.6%, G 9.3%, C 14.0%, T 37.1%). The phylogenetic relationships indicated that the species of S. *gracilior* emerged as sister to *Sarcophaga melanura*. This study provides important mitochondrial data for further studying evolutionary relationships and species identification of flesh flies.

The Sarcophagidae family plays a significant role in epidemiology and medicine (Ren et al. 2018). *Sarcophaga gracilior* Chen, 1975 (Diptera: Sarcophagidae), as known as *Tricholioproctia (Hamimembrana) gracilior*, has been recorded only in China and Nepal (Pape 1996). Here, we present the complete mitochondrial genome (mitogenome) of *S. gracilior*, which would further enrich our understanding of the phylogenetic relationship of *Sarcophaga* genus (Cameron 2014).

In this study, adult specimens were captured by pig livers in 4 August 2020, Guiyang city (25°46′N, 112°43′E), Guizhou province, China. All specimens were identified by traditional morphological keys, and then these specimens were stored at −80 °C in Guo’s lab (Hunan, Changsha, China) with a unique code (CSU2021012101). According to the manufacture's instruction, total DNA was extracted from thoracic muscle tissues of an adult specimen using QIANamp Micro DNA Kit (Qiangen Biotech Co., Ltd., Beijing, China). The mitogenome sequencing of *S. gracilior* was performed on an Illumina HiSeq 2500 Platform (Illumina, San Diego, CA), and then *de novo* assembly was carried out with MITObim v 1.9 and SOAPdenovo v2.04. Finally, the rough boundaries of all genes were initially identified by the MITOS2 Web Server (http://mitos2.bioinf.uni-leipzig.de/index.py) (Bernt et al. 2013).

In this study, the mitogenome of S. *gracilior* was first sequenced, which was 15,534 bp in length (GenBank No. MW531675), containing 13 protein-coding genes (PCGs), two ribosomal RNAs (rRNAs), 22 transfer RNAs (tRNAs), and a non-coding control region. The arrangement of genes was identical to that of ancestral metazoan (Cameron 2014). Nucleotide composition revealed a highly A + T bias, accounting for 76.7% (A 39.6%, G 9.3%, C 14.0%, T 37.1%).

Phylogenetic analysis of *S. gracilior* with nine sarcophagids species was constructed using the maximum likelihood (ML) method based on the 13 PCGs, and *Calliphora vomitoria* (Diptera: Calliphoridae) was used as an outgroup (Figure 1). ML analysis was performed by IQ-TREE v1.6.12 (Ren et al. 2020). In the phylogenetic tree, the species-level relationship between *S. gracilior* and *Sarcophaga melanura* closely clustered together, and separated clearly from the rest of species. The branches were well supported. This study provides significant mitochondrial data for further studying on evolutionary relationships and species identification of flesh flies.

**Figure 1.**
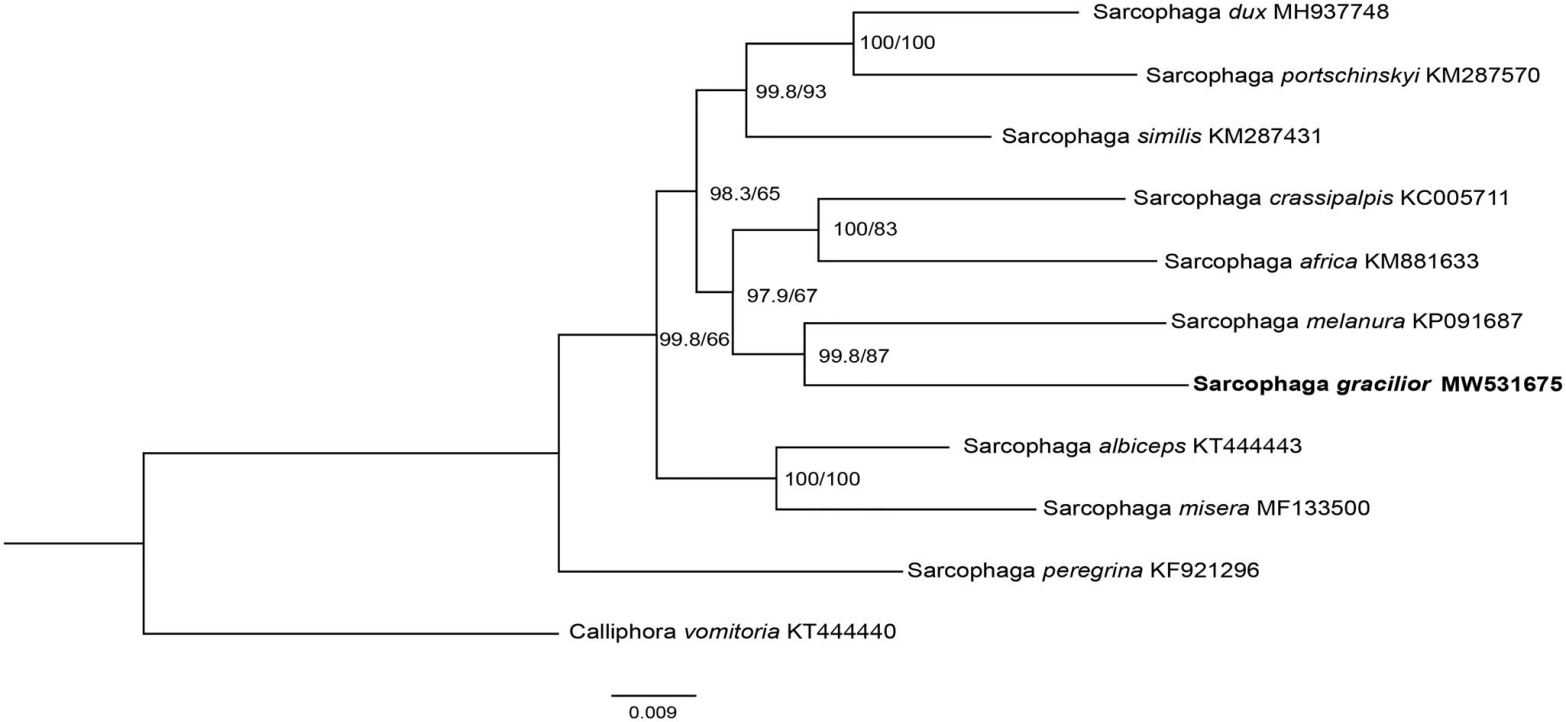
Phylogenetic trees of *S. gracilior* with nine sarcophagids species based on 13 PCGs by maximum-likelihood (ML) method. *Calliphora vomitoria* was selected as an outgroup.

## Data Availability

The data that support the findings of this study are openly available in NCBI at https://www.ncbi.nlm.nih.gov (GenBank: MW531675, BioProject: PRJNA695801, BioSample: SAMN17676972, SRA: SRR13608376). The sample was stored in Guo’s laboratory (Yadong Guo PhD, gdy82@126.com) with a unique code CSU2021012101.
